# Co-Culturing Human Adipose Derived Stem Cells and Schwann Cells on Spider Silk—A New Approach as Prerequisite for Enhanced Nerve Regeneration

**DOI:** 10.3390/ijms20010071

**Published:** 2018-12-24

**Authors:** Annika Resch, Sonja Wolf, Anda Mann, Tamara Weiss, Alexandra-Larissa Stetco, Christine Radtke

**Affiliations:** 1 Division of Plastic and Reconstructive Surgery, Department of Surgery, Medical University of Vienna, Spitalgasse 23, 1090 Vienna, Austria; annika.resch@meduniwien.ac.at; 2 Experimental Laboratory of the Division of Plastic and Reconstructive Surgery, Department of Surgery, Medical University of Vienna, Spitalgasse 23, 1090 Vienna, Austria; sonja.wolf@meduniwien.ac.at (S.W.); anda.mann@meduniwien.ac.at (A.M.); tamara.weiss@meduniwien.ac.at (T.W.); alexandra-larissa.stetco@meduniwien.ac.at (A.-L.S.)

**Keywords:** nerve reconstruction, cell–material interaction, nephila, co-culture, 3D-scaffold, regenerative medicine

## Abstract

Fast recovery is crucial for a successful nerve repair and an optimal functional outcome after peripheral nerve injury. Regarding donor site morbidity, autologous transplantation shows great limitations, which urge the need for alternative options in nerve reconstruction. Spider silk was reported as an advantageous material for cell adhesion, migration and proliferation, and its use in conduits is of great interest, especially in combination with cells to improve nerve regeneration. We here described the behavior of a co-culture of human Schwann cells and human adipose-derived stem cells (ADSCs) on spider silk as a new approach. After characterized by immunostaining ADSCs and Schwann cells were seeded in the co-culture on a spider silk scaffold and observed for 21 days. Results showed that cells were attached to the silk and aligned along the silk fibers. With further culture time, cells migrated along the silk and increased in number and formed an almost confluent cell layer. In immunostaining, results suggest that the cell layer was equally composed of ADSCs and Schwann cells. In conclusion, we showed that by providing a guiding structure for directed growth and cells to support nerve regeneration and remyelination, a valid alternative to autologous nerve grafts could have been found.

## 1. Introduction

Peripheral nerve injury is an important and common cause of patient long-term disability and decreased function [1,2]. Fast and undisturbed healing is crucial for an optimal regeneration after peripheral nerve injury. However, even after immediate nerve repair, clinical results are often disappointing with reduced sensory and functional recovery [3,4,5]. Moreover, direct axonal coaptation is limited to small gaps and impaired by scarring and neuroma formation [4,6,7].

Innovative options for nerve reconstruction after peripheral nerve injury are of great interest in plastic and reconstructive surgery. Treatment of nerve defect injuries by autologous nerve transplantation represents the gold standard when a tension-free end-to-end coaptation is not achievable. However, with regard to donor site morbidity, nerve availability is limited [2,6,7,8,9].

In the last years, many studies focused on finding valid alternatives. Nerve conduits made of biodegradable materials were developed as guiding channels for regenerating axons and redirect proximal nerve growth [2,4,6,7,8,9,10]. In some cases, conduits were filled with hydrogels or similar materials or neurotrophic factors to further improve and enhance recovery in peripheral nerve gap injuries. It was demonstrated that by applying intraluminal fillers, the regeneration was superior to empty conduits [1,2,11].

Moreover, seeding these conduits with cells, such as Schwann cells or stem cells, can further improve recovery and create a beneficial environment for nerve regeneration [11,12]. Schwann cell migration and proliferation represents the key to successful nerve repair, because they produce essential trophic factors, chemokines, extracellular matrix and serve as guiding structures that promote axon regrowth [1,3,13,14]. Since clinical use of isolated Schwann cells is limited due to donor site morbidity and slow growth in vitro, alternative sources of easy accessible cells are needed [3,5,15,16]. Cell properties, such as high proliferation rates in vitro, multipotent differentiation and integration into host tissue, are required [11,15,17]. Adipose-derived stem cells (ADSCs) have been identified as a viable alternative. Compared with other stem cells, ADSCs can be harvested by less invasive procedures (e.g., liposuction) and easily cultured with a greater proliferation rate in vitro. As shown in many studies, ADSCs possess the potential to differentiate into several functional cell types (e.g., adipocyte, osteoblast, chondrocyte and neural phenotypes [18,19]) and are, therefore, of high interest for cell-based therapies and clinical application [12,15,18,20,21]. Additionally, ADSCs secret multiple growth factors and cytokines (e.g., vascular-endothelial-growth factor-1, insulin-like growth factors-1, epidermal growth factor, hepatocyte growth factor, keratinocyte growth factor, angiopoietin-1, stromal cell-derived factor-1, macrophage inflammatory protein-1a and -1b and erythropoietin [20]) and provide immunomodulatory functions, which might further support and enhance the regeneration process of injured nerve axons [3,20,22,23,24].

Dai et al. [13] demonstrated that the best functional recovery after sciatic nerve gap injury was achieved by introducing a co-culture of Schwann cells and stem cells into conduits; however, in his experimental setup, no additional filler material was used. Constructs made out of silk have already been tested successfully in nerve defect injuries as nerve implants [9]. The use of spider silk could provide an advantageous guidance tool to improve regeneration after peripheral nerve injury [2]. With its biocompatibility, it does not need any modifications to its applications. Spider dragline silk can be easily harvested and the silk fibers are degradable by proteolytic degradation without pH changes. The latter is important, as pH changes could have potentially unfavorable effects on axonal regeneration and remyelination [4,25]. Spider silk possesses remarkable mechanical properties such as a tensile strength of 1.1 GPa, as well as an elasticity up to 35% with an average weight of 1.3 g/cm^3^ and an estimated average diameter of 1.0–4.0 µm. It shows a thermal resistance from 75 to 230 °C. The structure of the silk is composed of heavy-chain fibroin with cumulated regions of glycine and alanine amino acid sequences. This characteristic structure may contribute to the mechanical properties [9,10].

In recent years, studies using Schwann cells isolated from rats seeded on spider silk showed positive results concerning proliferation and regeneration rates [4,6,9]. Hence, taking all of these findings together, seeding Schwann cells and ADSCs as a co-culture on a guiding structure, like spider silk, may lead to an optimal environment for axonal regeneration by combining advantageous of both cell lines and beneficial properties of the silk.

In our study, we aimed to examine the behavior of a co-culture consisting of human ADSCs and human Schwann cells on a spider silk scaffold, regarding their proliferation and migration properties as well as their cell-to-cell interaction in the co-culture on spider silk.

## 2. Results

### 2.1. Immunostaining Confirmed Human Schwann Cell and Human ADSC Identity

**Figure 1:**
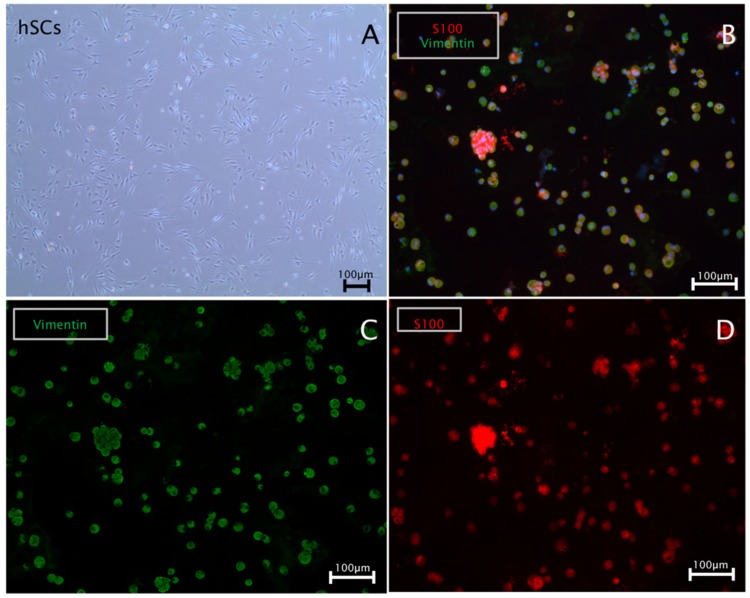
Characterization of human Schwann cell cultures: (**A**) a bright field image of a Schwann cell culture in vitro; (**B**–**D**) a representative immunostaining of human Schwann cell cytospins; (**B**) positive co-staining for S-100 (red), vimentin (green) and 4′,6-Diamidin-2-phenylindol (DAPI) (blue); respective single channels for (**C**) vimentin (green) and (**D**) S-100 (red).

Schwann cells were cultured in 6-well plates until a confluency of 90% was reached. During the spitting procedure, a cell aliquot was used for cytospins to validate the expression of Schwann cell marker S-100 and the intermediate filament vimentin by immunocytochemistry. Results showed highly pure Schwann cell cultures positive for S-100 and vimentin (Figure 1).

**Figure 2:**
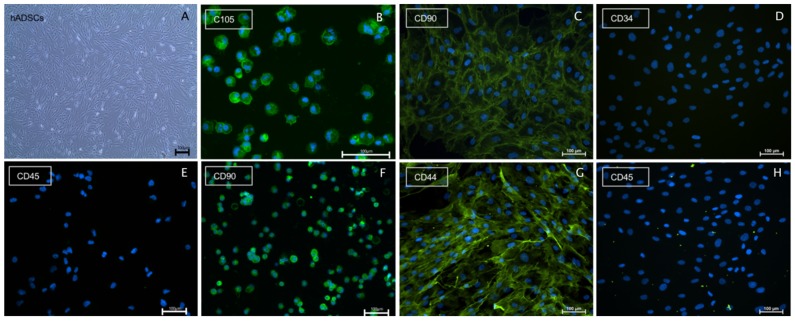
Characterization of human ADSC cultures. Scale bars in (**A**–**H**) represent 100 µm: (**A**) a bright field image of an ADSC culture in vitro at 80% confluency; (**B**–**H**) representative immunostainings of human ADSCs cytospins (**B**,**E**,**F**) or grown in vitro cultures (**C**,**D**,**G**,**H**); (**B**) CD105 positive immunostaining of ADSCs cytospin; (**C**) CD90 positive immunostaining of grown ADSCs cultures; (**D**) CD34 negative immunostaining of grown ADSCs culture; (**E**) CD45 negative immunostaining of ADSC cytospin; (**F**) CD90 positive immunostaining of ADSC cytospin; (**G**) CD44 positive immunostaining of ADSCs in culture; and (**H**) CD45 negative immunostaining of ADSCs cultures.

ADSCs were cultured in a T75 flask for 3 days until 80% confluency was reached. Prior to seeding on the spider silk, cell aliquots were routinely cytospined to characterize cells using immunocytochemistry. ADSCs were stained with a specific marker panel, confirming positivity for reported ADSC surface molecules CD105, CD90, CD44 and negative staining for CD34 and CD45 as a control, illustrated in Figure 2.

### 2.2. Spider Silk Enables Successful Adherence, Migration and Cell Expansion of Co-Cultures Composed of Human Schwann Cells and ADSCs

**Figure 3:**
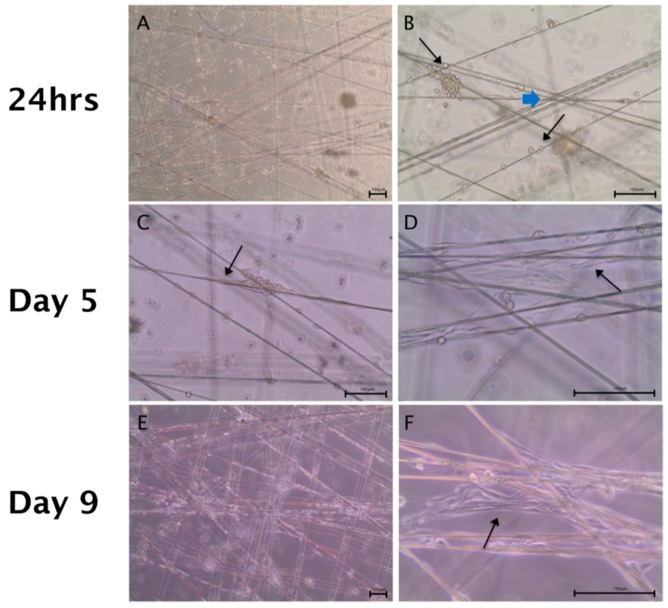
ADSCs and Schwann cells in co-culture on a spider silk scaffold from day 1 to day 9. Scale bars in (**A**–**F**) represent 100 µm. Bright field images illustrate co-cultures in low (**A**,**C**,**E**) and high (**B**,**D**,**F**) magnifications, respectively. (**A**,**B**) On day 1, cell attachment on the silk was observed (indicated by black arrows in (**B**)). (**C**,**D**) After 5 days in culture, cells had elongated along the silk and accumulated at fiber crossing (indicated by the blue arrow in (**B**)), resulting in a 3-dimensional organization of the cells. (**E**,**F**) After 9 days, cells were distributed along the silk fibers which were used as guidance structures and started to bridge gaps between the silk fibers (indicated by black arrows in **C**,**D**,**F**).

The co-culture of human Schwann cells and ADSCs was seeded on the spider silk fibers, as described in the methods part. Already within the first 24 h after the seeding, early cell attachment on the silk fibers was observed (Figure 3A,B). After 5 days in culture, cells started to elongate along the silk, and we observed a preferential accumulation of cells in the area, where the fibers crossed. Cells migration started from the corners, resulting in a 3-dimensional organization of the cells (Figure 3C,D). After 9 days, cells migrated and spread along the silk fibers, which were used as guidance structure, starting to form cell connections that bridged gaps between the silk fibers (Figure 3E,F, black arrow).

**Figure 4:**
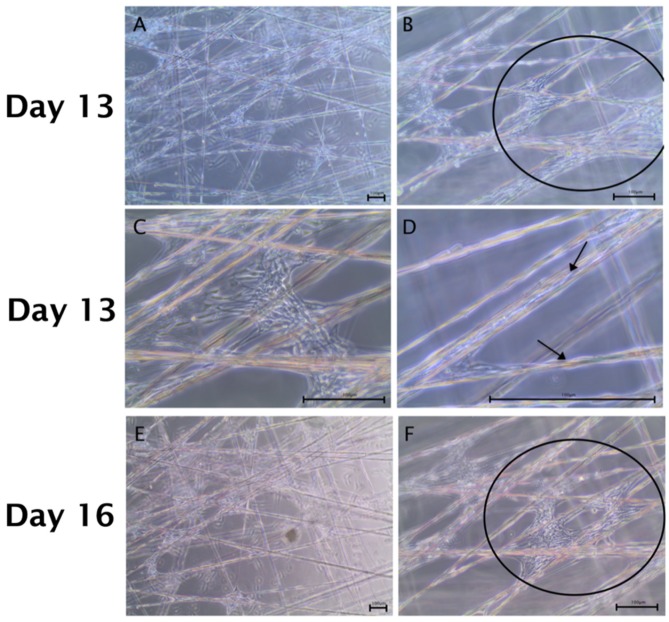
ADSCs and Schwann cells in co-culture on a spider silk scaffold from day 13 to day 16. Scale bars in (**A**–**F**) represent 100 µm. Bright field images illustrate co-cultures in low (**A**,**C**,**E**) and high (**B**,**D**,**F**) magnifications, respectively. (**A**–**D**) By day 13, cells had distributed equally along the silk fibers and confluent cell layers were observed at fibers crossing (indicated by the black circle in (**B**)); (**D**) cells migrating along the silk showing long shaped morphology (indicated by black arrows in (**D**)). (**E**,**F**) After day 16, an almost confluent layer of cells was formed over the mesh (shown in the black circle in (**F**)).

By day 13, the cell number was substantially increased and cells were equally distributed along the fibers with confluent cell layers being present at fiber crossings. With increasing cell proliferation, not only the cell density would rise along the silk fibers, but also cells stretched across the squares. Cells migrating along the silk showing long shaped morphology (Figure 4A,D). An almost confluent layer of cells was formed after 16 days in culture (Figure 4E,F).

**Figure 5:**
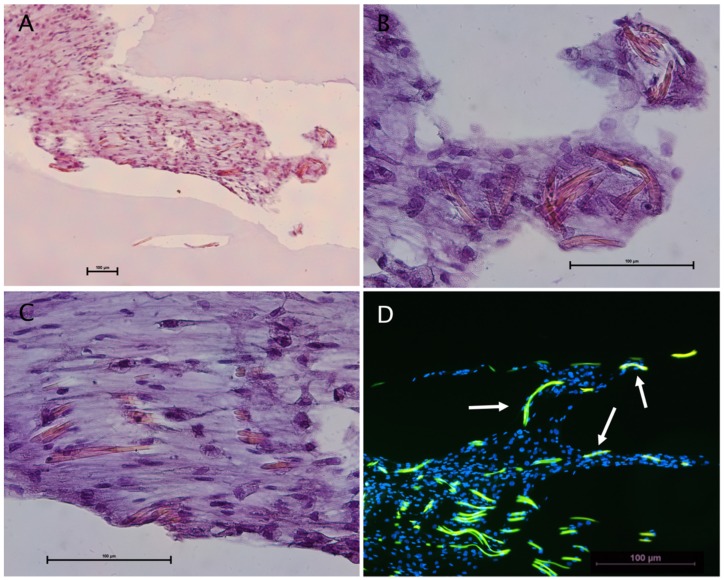
Illustrations of Schwann cells and ADSC co-cultures on spider silk: (**A**–**C**) Hematoxylin & Eosin staining showing cells equally distributed around the silk fibers; and (**D**) immunostaining with autofluorescence of the silk (green) and cell nuclei stained with DAPI (blue). Cells attached to the silk fibers are marked by the white arrows.

**Figure 6:**
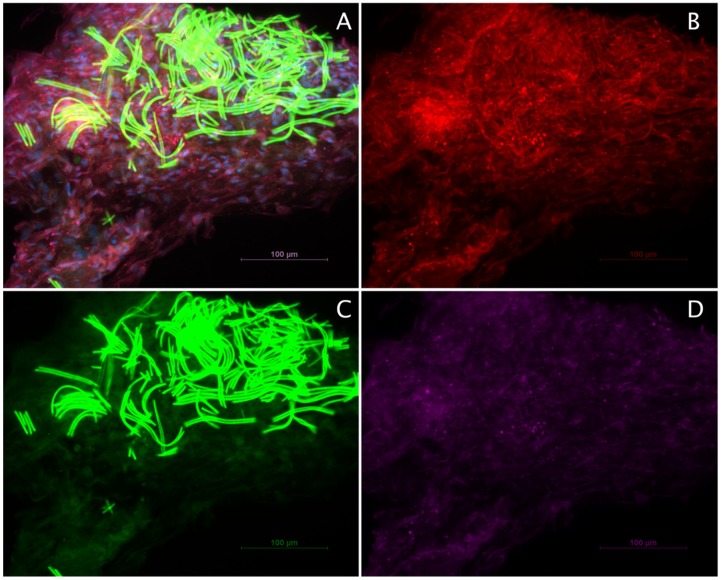
Characterization of Schwann cells and ADSC co-cultures on spider silk by immunohistochemistry. Scale bars in (**A**–**D**) represent 100 µm: (**A**) Representative image of paraffin sections stained for the characteristic Schwann cell marker S-100 (red), ADSC marker CD 90 (green), vimentin (purple), and DAPI (blue); (**B**) S-100 staining; (**C**) CD90 staining with strong autofluorescence of the spider silk fibers; and (**D**) vimentin staining.

After 21 days in culture, the construct of spider silk and cells was embedded in paraffin using HistoGelTM. Paraffin slides were cut and mounted onto microscope slides for analysis. Slides were either stained using the Hematoxylin & Eosin method (Figure 5) or used for immunohistochemistry triple-staining with S-100 as a characteristic Schwann cell marker and CD 90 as a marker for ADSCs, as well as vimentin, an intermediate filament present in both cell types (Figure 6).

The Hematoxylin & Eosin staining illustrated an equal cell distribution around the silk fibers (Figure 5A,C). Additionally, the immunostaining of the slides showed multiple cells attached along the silk fibers as well as surrounding the fibers, suggesting the cell adherence, migration and proliferation along the fibers was present (Figure 5D).

Immunostaining showed an almost homogenous positivity for all of the three markers, suggesting that ADSCs and Schwann cells migrated and proliferated equally along the silk fibers (Figure 6A–D). Notably, the silk possessed intense autofluorescence at 488 nm excitation (Figure 6A,C).

## 3. Discussion

For an optimal functional outcome after nerve gap injury, fast recovery of the injured nerve is indispensable. Autologous nerve transplantation states the gold standard of care when direct coaptation is not achievable. However, this method is largely limited by nerve availability and donor site morbidity. By developing a material comparable to autologous nerve grafts holding regeneration and remyelination properties, the problem and the limiting factor of donor site morbidity could finally be resolved. In the past, many scaffolds as an alternative to autologous nerve transplantation have been developed.

Materials should have the ability to enhance the healing process, be bioresorbable not disturbing the regeneration process [6]. A variety of biological materials, such as veins [26] and muscles [27] or constructions made out of biologic materials like collagen and fibrin [11], or synthetic materials such as silicone, have been studied for guidance of nerve defect repair [2,10]. Tested in various animal models, regeneration results were comparable to autologous nerve grafts in shorter distance nerve defects [1]. The attempt to introduce luminal fillers to these conduits was made primarily to enhance efficacy in shorter lesions but also found application in long-distance nerve defects to enhance nerve regeneration. In the review by Chen et al. [1], it was shown that a wide range of fillers have been used so far with some having a beneficial effect on nerve regeneration. Results suggest that besides the application of neurotrophic factors (e.g., nerve growth factor (NGF), and fibroplast growth factor (FGF)-1), which illustrated the most impressive effects and were cost-efficient, laminin-coated collagen and laminin-soaked sponge in conduits would lead to enhanced functional recovery in peripheral nerve gaps with increased myelinated axons and nerve potentials [1].

Further animal studies showed that the use of conduits seeded with cells achieved superior results in the functional analysis compared to blank conduits [13,24]. In the study conducted by Erba et al. [24] using a sciatic nerve model, they could show that axonal outgrowth from the proximal nerve stump could be stimulated by transplantation of ADSCs in an artificial nerve conduit. Furthermore, a greater Schwann cell migration into the conduits and proliferation in the distal stump could be observed compared to conduits without cells.

Our findings are supported by an animal model by Dai et al. [13], as the greatest functional recovery after sciatic nerve gap injury in this experimental setup was achieved by conduits seeded with a combination of Schwann cells and ADSCs as a co-culture. Results were evaluated by using the walking track, functional gait, nerve conduction velocity and histological analysis. The authors state that this may be due to an increased level of NGF compared to conduits seeded with Schwann cells and other cell types alone and blank conduits. These results are promoting the use of co-cultures in cell-based nerve regeneration research [13]. However, in contrast to our evaluations, no additional filler material was used in this study by Dai et al. [13]. By applying a filler material, a more advantageous environment for cell survival and proliferation can be provided by giving the cells the possibility of adherence and migration along a guiding structure. With this tool, cells will remain vital in the conduits over a longer period of time, giving the axons more time to grow inside the conduits and take advantage of the beneficial surrounding.

In this paper, we investigated the interaction between spider silk and a co-culture of human Schwann cells and human ADSCs on a manufactured scaffold of spider silk on a steal frame. Silk as a matrix for cell adhesion could further stimulate nerve regeneration by providing additional guidance to optimize axonal regrowth during regeneration and is, therefore, of great interest for research on nerve regeneration. With its unique properties, it may be possible to carry cells and guide tissue repair. As already shown in a previous study by Radtke et al. [4], spider silk can optimize directed axonal elongation. It was demonstrated that by inserting artificial nerve constructs made of veins filled with spider silk fibers in a long-distance peripheral nerve gap, Schwann cell migration, axonal regrowth and remyelination including electrophysiological recovery could be enhanced. Results showed that nerve regeneration was similar to autologous nerve transplantations. By additionally applying cells to the scaffold, a most optimal environment for nerve regeneration and axon elongation can be offered. In another study by Kornfeld et al. [9], in vivo tests demonstrated the viability of Schwann cells seeded on conduits inserted in long-distance nerve defects, with results indicating that the use of spider silk fibers with Schwann cells is promoting nerve regeneration and directed axonal regrowth.

The presented study goes one step further using a beneficial co-culture system composed of ADSCs and Schwann cells. In our research, we observed that starting from one drop of cells, a co-culture of Schwann cells and ADSCs adhered, expanded and migrated along spider silk fibers, which resulted in an almost confluent cell layer connecting the spider silk fibers. Staining of co-cultures for specific Schwann cell and ADSC markers illustrated an equal distribution of both cell types along and around the silk fibers. We were the first to present these data using human stem cells and human Schwann cells.

In summary, our results demonstrated that with cell attachment, migration and proliferation of ADSCs and Schwann cells on and along the silk fibers, a guiding structure for nerve regeneration can be provided by the spider silk. Hence, a promising tool and valid alternative to autologous nerve grafts for directed growth of the axons, with cells further supporting nerve regeneration and remyelination, could have been found. ADSCs can be easily obtained from a small amount of fat tissue and expanded rapidly in culture. By seeding ADSCs and Schwann cells in a co-culture, a smaller amount of Schwann cells is needed to still take advantage of their enhancing properties during nerve regeneration. Spider silk fibers represent a promising and suitable filling for peripheral nerve conduits and should be taken into consideration in nerve tissue engineering [4,7,9]. Pre-seeding silk fiber conduits with cells could have beneficial effects on the functional outcome even for long-distance peripheral nerve gaps and may thereby help to reduce and partly overcome the problem of donor site morbidity. To prove neuroregenerative potential of our findings, further experiments, control trials and ELISA for growth factor production as well as differentiation assays and in vivo testing are planned.

## 4. Materials and Methods

### 4.1. Isolation of Human ADSCs

Human ADSCs were isolated from the lipoaspirate of healthy patients after undergoing liposuction (Ethics committee Medical University of Vienna, 2079/2018, 11.12.2018). Harvested fat pads were mechanically dissociated and enzymatically digested by incubation with 1% BSA + 1 mg/mL collagenase Type 1, + 3 mM calcium chloride at 37 °C. Suspensions was first filtered through a gauze pad, and cells were then pelleted by 5 min of centrifugation by 220× *g*. Subsequent cell suspension was resuspended in the phosphate buffer solution (PBS) before centrifugation at 300× *g* for 5 min. Culture was maintained on 75 cm^2^ flasks in Dulbecco’s Modified Eagle Medium (DMEM) high glucose + 10% FCS + 1% Pen/Strep + 1 ng/mL human FGF and incubated at 37 °C.

### 4.2. Isolation of Human Schwann Cells

The human Schwann cells where isolated from nerves obtained in free flap surgery, when flaps were denervated (Ethics committee Medical University of Vienna, 2079/2018, 11.12.2018). The nerve specimen was first washed with PBS 1% antibiotic—antimycotic, and then transferred into αMEM + (αMEM + 2.5% HEPES, 1% Pen/Strep + 10% FCS + 1% NaPyruvat) for fascicular dissection. For further processing, fascicles were then transferred into a 6-well plate with 6–10 cm fascicle tissue each, incubated overnight on 37 °C with the digestion solution αMEM+ supplemented with 0.125% Collagenase Type IV, 1.25 U/ml Dispase II and 3 mM Ca_2_Cl_2_. After purification cells were seeded with a density of 2.5 × 10^5^ cells per well and cultivated in human Schwann cell expansion medim (hSCEM) (2% FCS, 1% Pen/Strep, 0.5% NaPyruvat, 2 μM Forskolin, 10 ng/mL hFGF, 10 ng/mL Heregulinβ1, 5 ng/mL PDGF-AA, and 0.5% N2 supplement). At the time of initial seeding, cells represented passage 0 (p0). Cells were seeded in Poly-l-Lysin (PLL)/laminin-coated 6-well plates. For the purification of the human Schwann cells, the two-step enrichment method was used. When cells showed a 80% confluency, the purification process was applied, exploiting the different attachment properties of the fibroblasts compared to Schwann cells [28].

### 4.3. Poly-l-Lysin/Laminin Coating

Six-well plates were coated using 0.01% PLL for 10 min at room temperature and let to dry. After 2 h, plates were incubated with 5 µg/mL laminin overnight at 37 °C.

### 4.4. Harvesting Spider Silk

Harvesting the spider silk fibers, we used adult females of the Nephilia edulis species. The spiders were fixed and immobilized carefully on a sponge with gauze and needles. For experimental practice, we used the major ampullate gland, which served the spider as security rope and building material. The major ampullate gland was stimulated by pulling the dragline out of the anterior spinneret mechanically. The fibers were pulled out slowly and woven on a steal frame until the density of the fibers was sufficient using a winding machine. The collected silk was woven on a steel frame and sterilized by autoclaving.

### 4.5. Seeding Co-Culture on Spider Silk

After characterization, the ADSCs and Schwann cells were seeded as a co-culture with 200,000 cells each on the spider silk construct on a steal frame and placed in a 6-well plate. The two cell types were mixed into a drop of 30 µL hSCEM media and then dropped gently onto the filaments. After letting them dry on room temperature for about 5 min, the scaffold with the co-culture was carefully put into the culture dish. After waiting for a few minutes, the 6-well was filled with hSCEM media until the steel frame with the silk was covered completely.

### 4.6. Cytospin Method

Cytospins were prepared for immunofluorescence staining following the protocol by Weiss et al. [28], and 8000 cells were applied per cytospin spun at 450× *g* for 7 min.

### 4.7. Immunofluorescence Staining

Paraffin sections were processed for immunofluorescent staining for detection of S-100, CD90 and vimentin. Slides were fixated with 4.5% formaldehyde for 15 min, and then blocked with 1% BSA in PBS + 7% Goat Serum. The primary antibodies were used as follows: First monoclonal CD90 (1:150 BD Pharmingen, Franklin Lakes, NJ USA) surface antibodies were added before cells were permeabilized with 0.2% Triton x in 1% BSA/1× PBS + 5% Goat Serum for 5 min. Subsequently, monoclonal S-100 (1:200 Dako, Glostrup, Denmark) and vimentin (1:300 Thermo Fisher, Waltham, MA, USA) as intracellular antibodies were added. Slides were then incubated overnight at 4 °C. Secondary antibodies were used as follows: anti-rabbit Alexa Fluor 594 (Abcam 1:300, Cambridge, United Kingdom), anti-mouse Alexa Fluor 488 (1:100 Thermo Fisher), anti-chicken DyLigh 650 (1:300 Thermo Fisher), and DAPI (1:500 Dako).

## Abbreviations

ADSC	adipose-derived stem cell
DAPI	4′,6-Diamidin-2-phenylindol
FGF	Fibroblast growth factor
PBS	phosphate buffer solution
DMEM	Dulbecco’s Modified Eagle Medium
PLL	Poly-l-Lysin
hSCEM	human Schwann cell expansion medium
